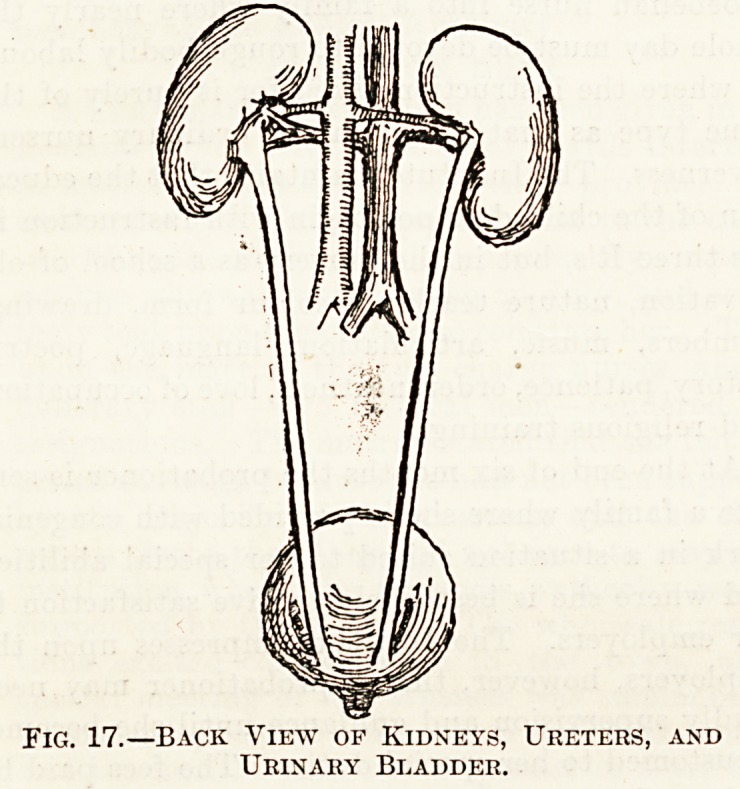# "The Hospital" Nursing Section

**Published:** 1906-08-04

**Authors:** 


### The Hospital.

**Nursing Section.**

Contributions for "THE HOSPITAL," should be addressed to the EDITOR, "THE HOSPITAL" NURSING SECTION, 28 & 29 Southampton Street, Strand, London, W.C.

No. 1,037.—VOL. XL. SATURDAY, AUGUST 4, 1906.

### Notes on News from the Nursing World.

### MIDWIFERY TRAINING IN POOR-LAW INFIRMARIES

THE good work done by the Central Midwives Board, which on Thursday last adjourned its sittings until October, has been freely acknowledged in our columns; but in one respect the Board has made a mistake. It has refused, without adequate reason, to recognise some of the leading Poor-law Infirmaries. Naturally, this policy has attracted the attention of the Local Government Board, and in the interests of its own important training schools has been resented. Intending probationers have, as it were, been warned off these schools because the Central Midwives Board has been more concerned about the number of cases attended by candidates for its certificate than about the manner in which the patients were treated. Under the new rules, therefore—which, we are officially informed, will not come into operation until February 12, 1907—the question of the training in midwifery in Poor-law Infirmaries will, at the instance of the Privy Council, be removed from the jurisdiction of the Central Midwives Board to that of the Local Government Board, who will thus be able to prevent the exclusion of institutions under their control from the benefits of the Midwives Act.

### NURSES AND EVIDENCE.

DURING the hearing of the inquiry into the mental condition of the Marquis Townshend, the male nurse who attended Lord Townshend gave evidence. Before he commenced, he appealed to the judge to allow his name to be suppressed, and Mr. Justice Bucknill consented. We can quite understand the desire of the nurse that his identity should not be proclaimed from the housetops, and, in a general way, we think that the interests of justice would be promoted if it were understood that the name of a nurse giving evidence would not be divulged in the face of any objection. It is very probable that nurses are often debarred from volunteering evidence because of their dread lest their names should be advertised to their prejudice. In the Townshend case an attempt was made in one paper to give the nurse the publicity not desired by means of an "illustration," but the likeness was so far from striking that we dare say it will defy recognition.

### KEEPING THE STAFF AT A MINIMUM,

THE Yeovil Town Council has given notice to the second nurse at the Isolation Hospital that her services are no longer necessary. At Chichester a proposal to increase the infirmary staff by the appointment of an additional nurse has been carried by the casting vote of the Chairman of the Guardians. Seeing that the number of inmates has risen this year to 116, as compared with 94 last year, and that many of the patients are always in bed or quite helpless, this addition seems to us to be essential. With regard to Yeovil, the reduction has been made because there are at present no cases in the Isolation Hospital. This sounds plausible, but in view of the size of Yeovil and of the forebodings which are entertained even now lest there should be a fresh outbreak of fever at this critical period of the year, we think it would have been wiser to retain the second nurse. Of course, the question of expense has to be considered; but keeping the staff at a minimum frequently means the sacrifice of efficiency and, in the long run, of economy.

### AN INQUIRY AT HOPE HOSPITAL.

THE Salford Guardians have very wisely decided that the allegations of mismanagement which have been brought against the authorities should form the subject of an inquiry by the Local Government Board. Whether true or false, the gravity of such charges as patients being kept short of food or clothing, or of being wiped with dirty sheets instead of towels, is obvious. It is only fair to mention that while these charges are put forward by an ex-attendant at the hospital, and have received some corroboration from a former sister, the Chairman of the Infirmary Committee states that he has in contradiction certain documents in his possession, signed by forty members of the staff now attached to the institution.

### HOLIDAYS AT THE COST OF EXTRA DUTY.

THE question of nurses' holidays has been engaging the attention of the Auckland Guardians. The medical officer recently stated in writing that the services of a special nurse were required during the absence of nurses on holiday. As showing the necessity of this appointment, he mentioned, in reference to a suggestion, that the nursing staff should take extra duty during the absence of any nurse on holiday, that of the seven nurses under the superintendent only one has been trained. He further said that, "considering the number of inmates in the hospital, and the number of nurses to deal with so many cases, to cause a nurse to take double or extra duty for a fortnight in order to get a fortnight's rest from work, would practically destroy all the benefit of the holiday to which she is entitled." In spite of this, the Guardians, acting on the advice of the Visiting Committee, have just determined to do without the assistance of an extra nurse. They must not be surprised if their persistence in the policy of making their nurses do extra

### HOW TO BECOME A NURSE

Many young women of the present day are, for one reason or another, attracted to Nursing as a career, but are at a loss to know how to enter the ranks of this important profession. "**How to Become a Nurse: The Nursing Profession, How and Where to Train,**" by Sir Henry Burdett, K.C.B. (price 2/4 post free), is the title of a most admirable work, which will be found to be a thoroughly reliable guide to this noble calling. It contains a list of the recognised Training Schools throughout the English-speaking world, with particulars respecting Hospital, Infirmary, District, and Military Nursing, and answers very completely all the enquiries of aspirants who would fain believe that they have a vocation for Nursing.

It is well, however, before entering upon a Probationership, to obtain some knowledge of the duties of a Nurse. These are very fully explained in "**Nursing: Hints to Probationers on Practical Work,**" by Mary Annesley Voysey (price 2/3 post free). From this book a very clear insight may be gained into the character of the training it is necessary to undergo, and of the qualities that are absolutely essential to success in this self-sacrificing profession.

Having decided to adopt Nursing as a profession, it would be advisable, whilst waiting for a vacancy in the Institution of your choice, or in the early stages of your probation, to acquire some knowledge of the elements of nursing, bandaging, and the structure and working of the human body. In this direction, "**Nursing: its Theory and Practice,**" by Dr. Percy Lewis (3/6 post free); "**A Practical Guide to Bandaging and Dressings,**" by Dr. Johnson Smith (2/- post free); "**Elementary Physiology for Nurses,**" by Dr. Marshall (2/- post free); "**Elementary Anatomy and Surgery for Nurses;**" by Wm. McAdam Eccles, M.S. (2/6 post free); and "**Elements of Anatomy and Physiology,**" by Dr. W. Bernard Secretan (2/3 post free), will be found most useful, and the information they convey is clear, concise, and accurate.

It is of course impossible to avoid the use of technical terms in such works as these, and it is therefore necessary that a good dictionary should be at hand for constant reference. "**The Nurse's Pronouncing Dictionary,**" by Honnor Morten (2/- post free), has been compiled especially for the use of Nurses, and contains the definition and pronunciation of most of the terms used in Medical and Nursing treatment. Its size also is a strong point in its favour, as it can very easily be carried in the apron pocket.

Another most useful work, and one it would be well to obtain, is "**Surgical Instruments and Appliances,**" by Harold Burrows, F.R.C.S. (1/8 post free). This book is of the utmost value to the Probationer, as by its assistance she will be able to readily identify the instruments and appliances used in various operations.

Many other important works on Nursing and kindred subjects will be found in the catalogue of The Scientific Press, Ltd., who give almost exclusive attention to the production of Nursing Textbooks; and the fact that their publications are in use in the foremost Training Schools is indisputable evidence of their value.

The Scientific Press, Ltd., 28-29 Southampton Street, Strand, London, W.C., will be pleased to send you, free of cost, on receipt of post-card, a copy of their latest Catalogue of Nursing Manuals, Charts, Case-Books, &c.

duty to obtain a holiday results in the breakdown of some of the staff.

### THE MILITARY NURSING SERVICE.

Miss M. McKenna and Miss J. H. Congleton have been appointed staff nurses in Queen Alexandra's Imperial Military Nursing Service. Miss E. B. Darnell, staff nurse, has been transferred from the Royal Arsenal Hospital, Woolwich, to the Queen Alexandra Military Hospital, Millbank. Miss M. E. Medforth, and Miss I. J. Pooley, staff nurses, have been appointed to the Royal Infirmary, Dublin; and Miss E. C. Peacock, staff nurse, to the Connaught Hospital, Aldershot. The appointments of Miss J. S. G. Gardner, Miss M. S. Williams, and Miss M. D. Woodhouse as staff nurses have been confirmed.

### IMPORTANT APPOINTMENTS TO THE SOUTH-EASTERN FEVER HOSPITAL.

The reopening of the South-Eastern Hospital at New Cross after rebuilding has been marked by the appointment of a large number of charge nurses. We announce the names and qualifications of nineteen on another page, and we understand that others will be selected later. The list as we give it is interesting, and affords a striking proof of the care taken to choose nurses who have been well trained. Among the schools in which the new charge nurses were trained may be mentioned Guy's Hospital, the Royal Free Hospital, the Middlesex Hospital, the General Hospital, Birmingham, Kensington Infirmary, East Dulwich Infirmary, Crumpsall Infirmary, and the Royal Victoria Hospital, Belfast. One charge nurse has been sister at Croydon Poor-law Infirmary; another at the Union Hospital, Sunderland; a third at the Fir Vale Infirmary, Sheffield; and a fourth at the Poor-law Infirmary, North Evington, Leicester. According to the details supplied, only three appear to have had experience in fever hospitals.

### SWIMMING COMPETITIONS BY NURSES.

The Guy's Hospital Swimming Club gave an entertainment at the Southwark Baths on Thursday evening last week. About forty of the nursing staff were present, and some of the staff and students. The spectators watched the performance from the surrounding balcony. The frequent applause suggested proficiency, and the plunging and diving competitors certainly gave the judges a difficult task. Four nurses entered for a sixty-yards race, the prize being won by Nurse Weber. One of the competitors was of Swedish and another of Danish nationality. Sir Alfred Fripp, the President of the Club, assisted in the starting, and Lady Fripp gave away the prizes at the close of the performance. A vote of thanks was passed to Miss Swift, the matron, for allowing the nurses to compete, thus adding to the interest and success of the evening.

### THE NURSES' UNION.

At Friedenheim Hospital last week an "At Home" was held for the members of the Nurses' Union. About fifty nurses attended, and, in addition to Miss Davidson, matron of Friedenheim and hostess of the occasion, there were present Miss Dashwood, Hon. Secretary of the Union; Miss Little, matron of St. Mary, Islington, Infirmary; Miss Vernet and Miss Bogg, of Middlesex Hospital. Tea was served on the lawn under the shade of the trees, and Dr. Lush, one of the hon. medical officers, gave an interesting address on "The Motives for Nursing the Sick." Miss Dashwood and Miss Davidson also spoke briefly. The hospital was thrown open to visitors, and most of the nurses availed themselves of this privilege.

### CUMBERLAND INFIRMARY.

Some years ago the Committee of the Cumberland Infirmary at Carlisle adopted the four-years' course of training for probationers. The first two and a half years are spent in the wards of the hospital, then six months' training is taken by arrangement at Plaistow Hospital, and the fourth year is spent on the private nursing staff. Before a certificate can be obtained each nurse must attend courses of lectures on Elementary Anatomy and Physiology, given by the resident medical officer; on Practical Nursing in the Wards, by the matron; and on Medical and Surgical Nursing, by the assistant physician and assistant surgeon. The examinations for the past year have just been concluded, and Nurse M. E. Atkinson has gained the Chairman's silver medal and the matron's prize for junior nurses. Nurse J. L. Shields obtained the Chairman's medal in the senior nurses' examination.

### LADY DUFFERIN ON DISTRICT NURSING.

The Dowager Marchioness of Dufferin presided at the annual meeting of the Bangor District Nursing Society, and, in submitting the report, referred to the great necessity existing in Bangor for a fully-trained maternity nurse. The Society, she said, was ready to take the matter up when its members received sufficient moral and financial support to make the undertaking possible. Lady Dufferin alluded in warm terms to the work of the nurses who had attended 227 cases, and to the usefulness of the Samaritan Fund. An address was then delivered by Professor Byles on "Infantile Mortality," and Lady Dufferin subsequently expressed her high appreciation of its value and her thanks to him for coming to deliver it.

### THE NEW HOME AT READING.

There was an interesting ceremony at Reading on Thursday last week when the new Home for Nurses in connection with the Queen Victoria's Nursing Institute was opened. The Home, which is situated in Abbot's Walk, has, in addition to the usual offices in the basement, a disinfecting-room, a store for apparatus, and a cycle-house, which has a separate approach from outside. The rooms on the ground floor comprise an office, a private apartment for the lady superintendent, a waiting-hall, and a dining-room; in the first floor is a handsomely furnished sitting-room with a front balcony, and above are ten bedrooms with ample bath-room accommodation. The numerous guests at the opening function were received by the Mayor of Reading, who afterwards addressed them, and called upon the Honorary Secretary to make a statement of the position of the Institute. In this statement it was mentioned that Messrs. Sutton and Sons intended to give an annual subscription equivalent to the rent of the house hitherto occupied by the nurses, so that the Institute will not suffer financially by the relinquishment of the former Home. Mrs. Sutton having unveiled the stone set in front of the building, and declared the latter open, Mr. G. W. Palmer proposed a vote of thanks to her, and Mr. Harold Boulton seconded the proposition in a speech of some length, making suitable reference to the good work done by the Queen's nurses in the town. Mr. Sutton, returning thanks on behalf of his wife, alluded to the excellent services rendered by Miss Carter, the lady superintendent, and the members of the Ladies' Auxiliary.

### ASLEEP ON DUTY.

A remarkable illustration has been given in New Zealand of the folly of hospital boards interfering in matters which are outside their province. The trustees of the Waihi Hospital came to the conclusion that the matron had unnecessarily suspended a nurse who was found asleep on duty, and accordingly took upon themselves to reinstate her. Thereupon the matron, the two charge nurses, and the honorary staff—two medical men—tendered their resignations. The matron stated that she felt compelled to resign because she had not been supported by the majority of the trustees, and the charge nurses wrote that they did not care to stay in an institution where the matron's authority was not supported by the Board. The wholesale resignations excited consternation in the town, and a special meeting of the trustees was summoned, at which an appeal to the matron and charge nurses and the medical men not to persist in their determination, was adopted. Many complimentary remarks were also made in the speeches at the meeting; and, in spite of some opposition, the fact was placed on record that the action of the Board in reinstating the nurse who fell asleep on duty "was not intended in any way as a reflection on the matron." Of course, the Board were quite in the wrong. It is a very serious matter for a nurse to go to sleep while she is on duty. The cause must be either ill-health, in which case the nurse ought not to be on duty; or laziness, in which event she should be strongly reprimanded. Moreover, in this instance the nurse refused to apologise for her breach of duty. The intervention of the Board was a distinct affront to the matron, who treated it, as the sequel shows, in the most judicious manner possible.

### A VACANCY THAT WAS NOT FILLED.

The Lewes Guardians recently advertised for a nurse to fill a vacancy on the Infirmary staff. Several applications were received, and three candidates were selected for an interview with a Committee. The Committee assembled on the date specified, and a conveyance was in readiness at the Union offices in Fisher Street, Lewes, to take the nurses to the Workhouse at Chailey. One of the three failed to turn up; another, finding no carriage at the station to meet her, returned forthwith to London; and the third hired a cab and drove to the Workhouse. She was offered the appointment, and apparently seemed gratified, but a few days later wrote to say that she was unable to accept it. A further selection of candidates has since been made, and we hope that on the second occasion the Lewes Guardians may be more fortunate. It might be worth while to send the conveyance to the station.

### A STEP IN THE RIGHT DIRECTION.

According to a Local Government Board Order in the *London Gazette* of Tuesday, the superintendent nurse at Chelmsford Union Workhouse supersedes the master and matron in the duty of visiting and supervising the sick and lying-in wards.

### SEASIDE AND HOLIDAY APARTMENTS.

For the assistance and convenience of our readers during the holiday time, the manager of The Hospital has started a special column for advertisements of seaside and holiday apartments. We have no doubt that many nurses will be glad to be saved, by this means, the trouble of either wading through the daily papers, writing to friends for recommendations, or taking the always perilous course of making off to popular resorts in the height of the season without securing comfortable quarters in advance. Those who are only able to be away for a brief period, and perhaps merely for Bank Holiday, may be glad to be reminded that the New Palace Steamers make their usual daily sea trips down the river Thames to Margate, Ramsgate, Deal, and Dover. The accommodation provided is so excellent, and the expense is so small, that in the present state of the weather these trips are sure to recommend themselves to nurses who desire to enjoy a taste of sea air.

### A TWENTY-THOUSAND SHILLING FUND.

The happy notion occurred to the official of the Cardiff branch of Queen Victoria Jubilee Institute to originate, under the title of the Marchioness of Bute's Twenty Thousand Shillings Fund, a movement for the financial assistance of the Maternity Home, which is very much in need of proper equipment. So far the response has been the subscription of upwards of seven thousand shillings. This includes a number of smaller contributions, which are gratefully received. Some of the very poor patients have sent sixpences, with expressions of regret that they could not do more. It need hardly be added that larger sums than a shilling are welcomed, but the main idea of the fund seems to have caught on in the town, and it is hoped that, with energy on the part of the collectors, the whole amount will be obtained.

### SHORT ITEMS.

Congratulations to Lieutenant-Colonel Bruce Morland Skinner, of the Royal Army Medical Corps, Secretary to the Advisory and Nursing Boards at the War Office, on his appointment by the King as a member of the Royal Victoria Order of the Fourth Class.—The Countess of Craven last week laid the foundation-stone of the Nurses' Home in connection with the Coventry and Warwickshire Hospital.—The Earl of Shaftesbury is organising a concert in Dublin during the horse-show week this month in aid of Lady Dudley's Fund for the Establishment of District Nurses in the West of Ireland.—The Indian Nursing Association is being well supported in India, and Lady Minto is now appealing for English subscriptions to her endowment fund.

### The Nursing Outlook.

"From magnanimity, all fears above;
From nobler recompense, above applause,
Which owes to man's short outlook all its charm."

### TRAINING FOR CHILDREN'S NURSES.

### II. The Norland Institute.

The Norland Institute, founded by Mrs. Walter Ward, has been working for over thirteen years with Miss Isabel Sharman supervising. The probationers' fees last year amounted to upwards of £4,500. Mrs. Ward's services have always been free, and she decided at the commencement to devote all the profits to the future benefit of the nurses. The nurses on the staff now number 421, and 64 probationers are at present in training. In the whole, 995 women have passed through the Institute, of whom upwards of 25 per cent. have withdrawn and about 17 per cent. have been dismissed. The Institute contains 32 beds for probationers, of which 30 are permanently full. Mrs. Ward experienced great difficulty at the outset in procuring recognition of the value and importance of careful nursery training as the foundation for the full development of the child at school. The work has had therefore an educational value; and it is now widely recognised as being ocnducted on sound principles essential to the well-being of small children in well-to-do families. The staff consists of one principal, two fully-qualified technical teachers, one lady cook, four Froebelian teachers, two experienced Norland nurses, and the secretary. There are also non-resident teachers and lecturers. The work entails a large correspondence, and about 8,000 letters were written last year. The applications for nurses far exceed the possibility of supply.

The training extends over a period of one year, commencing with 13 weeks in the Norland Institute, 13 weeks in a children's hospital, 10 weeks more at the Institute, and 10 weeks in practice in the nurseries, which, with six weeks' holiday, represents the whole year. The course of study comprises instruction in domestic work, under the superintendence of a fully-qualified technical teacher, who gives instruction in cookery, laundry work, and housewifery, the greater part of the household duties at the Institute being undertaken by the probationers. Lessons are given in the needlework required in the making and mending of simple garments, and in the cutting out and dressmaking for little children. Lessons are given also in the care and management of infants and young children, simple remedies and nursery diet. Probationers are trained in the care of healthy children at the Institute, and hospital experience is provided, because sick, convalescent, and incurable children can only be rightly cared for by those who have had practical teaching and experience. On her return from the children's hospital course the probationer receives instruction in Froebelian occupations and methods, nature study and brush work, the principles and art of education, and the care and management of little children.

The Institute aims at supplying ordinary nurses for manual work and the physical care of children only, and also nurses capable of caring for the moral and intellectual training of the children. The aim is to fit the nurses for both spheres, but it would be a sad waste of precious material to send a gifted Froebelian nurse into a family where nearly the whole day must be devoted to rough bodily labour, or where the instruction asked for is purely of the same type as that found in the ordinary nursery governess. The Institute maintains that the education of the child does not begin with instruction in the three R's, but in the nursery as a school of observation, nature teaching, colour form, drawing, numbers, music, articulation, language, poetry, history, patience, order, neatness, love of occupation, and religious training.

At the end of six months the probationer is sent into a family where she is provided with congenial work in a situation suited to her special abilities, and where she is best likely to give satisfaction to her employers. The Institute impresses upon the employers, however, that a probationer may need kindly supervision and guidance until she becomes accustomed to her special duties. The fees paid by each probationer, including the whole cost of instruction, uniform, hospital fees, teaching, board, residence, and washing are £74 8s. The minimum salary paid to a probationer at the end of six months is £20, but it is usually £24 for the first year, with an increase of £2 annually. At the end of three years' work the probationer is promoted to be a private nurse of the Institute, and she then is entitled to a considerable increase in her salary. The regulations provide that all Norland nurses working in families shall have proper provision made for meals, exercise, holidays, Sunday leave, and so forth. Nurses are not expected to scrub floors, clean grates, carry coals, to fetch and carry from the kitchen, or to wash clothes, except small woollen and dainty garments belonging to the children.

Probationers receive instruction in the nurseries of the Institute, which are arranged for little children from one month old up to seven or eight years of age, and are intended for the children of Indian officers and others on foreign service, widows, widowers, members of the theatrical profession, guardians, trustees, missionaries, colonials, or parents desiring a temporary safe and happy home for their children while they travel. The nurseries are shown to any persons interested who have any claim to the courtesy beyond one of mere curiosity. There can be no doubt that the work is well done, for it has been completely successful, is extending, and is likely to grow materially in the near future.

### The Care and Nursing of the Insane.

By Percy J. Baily, M.B., C.M.Edin., Medical Superintendent of Hanwell Asylum.

### I.—ANATOMY AND PHYSIOLOGY.

(*Continued from page* 231.)

### B. *The Urinary System*

comprises the kidneys with their ducts (the ureters), and the bladder and the narrow channel (the urethra), which carries the urine from the bladder (fig. 17). The function of the kidneys is to excrete the urine.

Fig. 17.—Back View of Kidneys, Ureters, and Urinary Bladder.

The kidneys are two in number—a right and a left one. They are firmly fixed to the back of the abdominal wall in the region of the loins, one on either side of the spinal column. They are of a characteristic shape—something like that of a bean seed, and weigh, as a rule, about four ounces each. The inner side of the kidney, that which is nearest the spinal column, is depressed or concave in shape, and is called the Hilus. Here a large artery enters the kidney, and a vein leaves it (the renal artery and vein), and from the same part the ureter proceeds from each kidney down to the bladder. The ureters are comparatively long and narrow tubes. They are in contact with the back wall of the abdominal cavity down which they pass into the pelvis, where they enter the bladder in its posterior or hind part, each one separately.

The bladder is an oval bag, which lies within the pelvis. It is lined inside with mucous membrane, and its walls contain a large amount of involuntary muscular fibres. At the "neck" of the bladder there is an opening which leads into the urethra, and which is surrounded by a ring of circularly arranged muscular fibres (involuntary) called the sphincter of the bladder.

The urine is constantly being excreted by the kidneys. From these glands it passes down the ureters into the bladder, which acts as a reservoir. It is retained in the bladder by the action of the sphincter muscle, which, when the bladder is full, may be relaxed by means of a nervous mechanism which is under the control of the will. The contents of the bladder then pass away through the urethra.

We have seen that carbonic acid, which is a gas, and is removed from the body by the lungs, is the result of the oxydation of carbon which exists in the food and tissues. Now, in like manner during the digestion and assimilation of nitrogenous foods, such as meat, fish, etc., and as the result of waste of our nitrogenous tissues, especially the muscles, certain solid waste matters are produced, the chief of which are urea and uric acid. These are removed from the blood by the kidneys, and are dissolved in the water of the urine. In addition to these the kidneys also remove from the blood various mineral salts.

The function of the kidneys, then, is the removal from the blood of

1. Water.
2. Certain solid waste matters, the chief constituent of which is nitrogen. These are urea and uric acid. Of the former about 500 grains, and of the latter 10 or 12 grains, are excreted in 24 hours.
3. Various saline matters, of which the chief are common salt and some phosphates and sulphates, chiefly of potassium and sodium.

Normal Urine is a pale yellow or straw-coloured clear fluid, of which about 50 ounces, or 2½ pints, are excreted in 24 hours. The amount, however, varies very greatly, according to circumstances, as we shall see more particularly in a later section. Its chief bulk is made up of water, which holds the other constituents in solution.

The following table shows its composition :—

| | | |
|---|---|---|
| Water. | | |
| Organic Waste Matters containing Nitrogen | Urea<br>Uric Acid | Combined with Pot. and Sod. forming Salts (Urates) |
| Inorganic Salts ... ... | | Common Salt (Chloride of Sod.)<br>Phosphates of Pot. and Sod.<br>Sulphates of Pot. and Sod. |

### C. *The Skin.*

The skin is a tough, elastic membrane which forms the outer covering of the body. It is separated from the deeper tissues by the fat. It consists of two distinct layers. The outer one—that is to say, the one in contact with the air—is the cuticle or scarf skin; the deeper one is known as the cutis vera or true skin.

*The Cuticle* consists of many layers of closely packed cells, and forms a more or less horny covering for and protection to the true skin. The cells immediately on the surface may be regarded as being dead, and they are constantly being brushed off into the air. They are replaced by the new cells, which grow and are being pushed up, as it were, from the deepest part of the cuticle, that, namely, which is in contact with the true skin. In health this loss of the superficial cells of the cuticle is not noticeable, but in many diseased states, after scarlet fever and erysipelas, etc., and in many forms of skin disease, the cuticle may peel off in flakes or scales of considerable size.

The cuticle contains no structures, there are no blood-vessels, and no nerves in it; but it is pierced, as we shall presently see, by the ducts of certain glands and by the hairs.

It serves a purpose which is purely mechanical, its only function being to cover and protect the vascular and highly sensitive cutis or true skin beneath it.

The *Cutis or True Skin* is abundantly supplied with blood-vessels and nerves. From it grow the cuticle and nails, and within it are contained the hair follicles from which the hairs grow, and two kinds of glands—namely, the sebaceous glands and the sweat glands.

The *Hair follicles* may be described as little pits or sacs in the cutis, from the bottom of which the hair grows.

The *Sebaceous Glands* are arranged about the hair follicles, into which their secretion is poured. This secretion is of an oily or greasy nature, and gives to the hair its natural gloss. It also serves to keep the cuticle soft, and prevents it from becoming dry.

The *Sweat Glands* secrete the perspiration. The body of the gland lies embedded within the cutis, but the duct pierces the cuticle as a minute spiral tube, and opens upon the surface. These little openings are present in thousands all over the body, and constitute what are often spoken of as the "pores" of the skin.

The sweat consists chiefly of water in which are dissolved some solids; of these one-half is common salt (sodium chloride). It varies enormously in amount from time to time, and is, under ordinary circumstances, imperceptible, since it evaporates from the surface as fast as it is secreted. In hot weather and during violent muscular exertion it is secreted much more rapidly than it can evaporate, and therefore it collects on the surface of the skin and forms beads of fluid.

Arterial blood, as it passes through the capillaries of the skin, is not rendered venous, but retains its arterial characteristics. Some carbonic acid is in fact given off by the skin, and a small amount of oxygen is absorbed from the atmosphere by the blood in the cutaneous capillaries.

On account of the large amount of blood contained in the skin a great deal of the heat of the body is lost through it. Hence the skin plays a very important part in the regulation of the temperature of the body.

Certain of the nerves of the body end in the skin. These nerve endings when stimulated give rise to certain impressions, which are conveyed by the nerves to the brain, and are there interpreted as various sensations, such as that of touch, pain, heat, cold (common sensation).

Briefly stated, then, the functions of the skin are these:—

1. It is a covering for the body.
2. It is an excretory organ, removing from the body water and salts, as well as a very minute quantity of urea.
3. It is, although in man to a very trivial extent, a respiratory organ.
4. It plays a part in the regulation of the temperature of the body.
5. It is the seat of common sensation.

Immediately beneath the skin there is a layer of fat which varies very much in thickness in different individuals. This, under normal conditions, forms a sort of cushion all over the body, fills up the hollows, and rounds off the angles, and adds plumpness and grace to the outline of the body. It prevents to a large extent the loss of heat, and acts as a reserve of nourishment in case of need.

### The Nurses' Clinic.

### NOSE AND THROAT CASES—DURING AND AFTER OPERATION.

During operation it is the nurse's duty to keep count of the number of swabs used and to watch that none are left in the nose, as they would cause sepsis, and probably purulent discharge. Also sponges used for the back of the throat must be carefully counted to see that none remain in the mouth.

A wide-mouthed bottle with solution should be ready to receive the tissues cut away. Probably a solution of formaldehyde would be used, but the nurse would receive orders as to that.

In adenoid operations the nurse should keep her eye on the gag to see that it does not slip out of position, or, if it does, that the lips do not get caught in when it is re-adjusted. In excision of the tonsil the cut portion must be removed at once, or it may obstruct breathing; as a rule it comes away with the guillotine.

It is the nurse's duty to keep the blood and mucus from going into the patient's eyes during an operation, as this would cause acute conjunctivitis. Some surgeons order moist swabs to be bandaged over the eyes, though this is not generally done, and the nurse can keep the eyes clear without that.

A set of tracheotomy instruments should always be in readiness in case of choking.

The nurse's duties after operation are very important. She may be called upon to meet emergencies, and should know how to act. She must be able to recognise and control complications which may arise after operation, when perhaps the surgeon is not on the spot. The most frequent complications are hæmorrhage, reaction, disturbance of parts, vomiting blood, headache, stupor, and sepsis. The temperature and pulse must be watched, and the diet administered regularly and carefully.

First, as the most important complication, we will take hæmorrhage.

Slight bleeding from the nostrils may occur without any danger, but if a continuous stream runs down the nose immediate steps should be taken to stop it. The nurse must examine the back of the throat, as, if a patient is lying down he will swallow the blood instead of it running down the nostrils. On examination of the throat, if hæmorrhage has started, it will be seen flowing freely down the operated side. Should the patient still be under the influence of the anæsthetic and hæmorrhage is suspected, roll him on to his side or abdomen, the head being lower than the shoulders, and if hæmorrhage has started it will flow from the mouth and nose; if this precaution is not taken the patient may swallow large quantities of

### THE NURSES' CLINIC—*Continued.*

blood, and suddenly become faint and white. In extreme cases of hæmorrhage transfusion may have to be resorted to. Nasal hæmorrhage may sometimes be stopped by pressing with the thumb the wing of the nose against the septum, with the head bent well forward. Steady pressure for five or ten minutes will stop very severe bleeding.

An ice-bag to the nape of the neck will help to arrest the bleeding also.

If after ten minutes, or a shorter time, the bleeding continues very severe, a cotton plug, hot-water douche, or complete plugging will have to be resorted to. A cotton wool plug is made of absorbent wool loosely wound on a bent applicator. This plug may be dry or soaked in peroxide of hydrogen solution. It is pushed directly backward the full length of the nostril, then the applicator is withdrawn, leaving the plug in, the end slightly protruding from the nostril. Should this be unsuccessful the plug must be withdrawn and styptics applied, as peroxide of hydrogen or hot-water douche (125° F.). The peroxide is sprayed into the nose.

To plug the nose well several swabs may be used (the nurse keeping count of how many have been inserted), or a long strip of gauze tightly packed in. The post-nasal tampon is the last resource. A nurse will rarely use it, but should know what to prepare for the surgeon. Several good-sized, firmly made tampons should be prepared, some larger than others, with a string tied round the middle, and two long ends left. A piece of rubber tubing or soft rubber catheter with a piece of string or tape looped running through it, the loop projecting three inches beyond the end of the tube, or eyelet-hole; if a catheter is used from the other end the loop should project the length of the tube. The tampon is usually an inch and a half by one inch in size. Some long strips of gauze should be also prepared.

Some antiseptic solution will be required to douche the nose with before plugging. This will possibly increase the hæmorrhage for a time, but renders the parts clean, and lessening the danger of sepsis arising from the plugs.

If, notwithstanding these measures, the bleeding persists, the plugs must be removed, and larger ones put in. Adenoid forceps are the most convenient for removing the plugs.

After plugging, reaction generally sets in, especially with very nervous patients. The symptoms are chilliness, headache, exhaustion and prostration, muscular pain in the legs and arms, nasal swelling, and discharge.

When reaction occurs the plugs must be removed and the wounds carefully cleansed with peroxide of hydrogen 1 in 20, followed by normal saline solution. Some drug will be ordered by the surgeon to allay the headache and fever. Iced cloths, applied to the forehead and changed frequently, keeping the parts very cold, will give great relief.

In cases where a nasal splint has been applied and gets out of position, it can be held up by a piece of adhesive plaster put over the protruding part and fastened to each side of the nose, till the surgeon can readjust it.

During an operation a quantity of blood may be swallowed and vomited afterwards, and may cause the nurse some alarm, but generally after one or two attacks of vomiting it will stop, and the patient's condition improve, but if the patient continues to be very collapsed it may be that hæmorrhage is going on from the wound, and the throat should be inspected.

If headache persists or gets more severe, and the eyes are sensitive to light, it should be reported at once, as there is always the danger of meningitis setting in.

Sepsis, "mild" or "severe," may occur, and may take the form of quinsy, tonsillitis, or follicular tonsillitis.

Sepsis is to be suspected if local inflammation, headache, pains in the back, and chilly sensations set in, and any of these signs should at once be reported.

Cleaning the nose after operation generally takes place in twenty-four hours. This, of course, is not done by the nurse. She should have some sterile or antiseptic solution ready for irrigation, also plenty of swabs on holders and forceps. Anything else required would be ordered.

After tonsillotomy the danger of sepsis is great, as it is impossible to keep the mouth sterile, but the nurse may do much to avoid this complication by irrigating the mouth frequently (three or four hourly). After twenty-four hours there is little danger of sepsis setting in.

After operation for cleft palate, the mouth must be frequently cleansed out with some sterile solution. The nose should also be irrigated.

Cleansing of the mouth by irrigation must be done with the patient lying on his chest, his face being over the side of the bed.

### Incidents in a Nurse's Life.

### IN A MATERNITY HOSPITAL.

It was just after midnight, the wards were quiet, the gas turned low, and many of the young mothers were asleep. The stillness of the summer's night only sometimes broken by the cry in the distance of one of the little newcomers down in the nursery. We were just finishing the "night round" and only one or two "waiting patients" remained to be visited before the house-surgeon retired for the night. It was with real regret that I said to her as we passed through to the next house, "This is the last time, doctor." We quietly went into the small ward where two expectant mothers were, one was asleep, so we passed her and went over to the other. Our gentle but reserved little doctor stooped down and asked Mrs. West why she too was not asleep. After a few minutes we moved to go, the doctor whispered with a smile, "Remember, you must come off by the morning." As we returned I said, "Do you think it possible, doctor?" "I wish it could be," she answered, "I should have liked to see it over before I go," and with a "good-night" to me she left me. I had little to do then and my thoughts would wander to the patient next door—waiting. I felt strangely nervous and full of unrest; I realised here was a case full of interest, one under treatment for some time by the senior house-surgeon whose six-months' course would end the following day, and watched with keen interest by her sister medical women connected with the hospital. This would be her second child; after a terrible fight some two or three years ago, she only had come out of it, and we hoped that treatment and skill would make her the mother of a living child, the doctor was leaving and it seemed without seeing the result of her care.

My unrest took me very often during the night to listen, although I was not entirely responsible for her then. At dawn I heard a well-known cry, and very hastily I went to Mrs. West, and in a few seconds she whispered with great joy, "You must take me over, nurse." I thought as I did so perhaps our doctor might be there after all. Some hours later when the outside world seemed hurrying to its work and the day nurses were already on duty, we had given up the night reports and were having dinner. A hurrying nurse brought the oft-repeated call to the labour ward, and as we ascended the stairs we met nurses coming from their various wards, all with one purpose, and it was whispered that it was Mrs. West this time! a nameless fear of what we were going to witness seemed to assail us all. What? Another tragedy, or our doctor's victory?

Silently we passed in, the medical women and students were already there, the doctor's face was very white as she sat a little apart. Nothing was heard save the moaning of the patient and now and then some whispered instruction to the student. We waited in breathless suspense, although we knew all was going well, when, thank heaven! the loud cry of her beautiful little son ended the silence, and as silently as we entered one by one the nurses filed out. We who remained heard a long-drawn sigh and the whisper "Doctor, I'm so sorry, I did try to be good." And I turned to the doctor and saw her eyes were filled with tears, as she quickly got up and left the ward. As I followed I said to myself "Ah! you are a woman after all, and this is your victory indeed."

### A Visit to a Dutch Hospital.

BY AN IRISH NURSE.

While spending a few days at The Hague recently, I was able to visit the Gemeente Hospital, the largest in the town.

It was quite a long drive over and along the canal; for, as my knowledge of the Dutch language is extremely limited, I did not venture to go by tram, and the distance being too great for me to attempt to walk, I took a cab, which gave me an opportunity of seeing something of The Hague.

The hospital is large, close to the canal, and built in the form of a square, with a garden and trees in the open space inside. In this square I saw through a window a number of patients sitting on comfortable chairs and looking very happy.

I had to wait for some time in a small room off the hall. I suppose they were looking for someone who could speak English, for the porter who took my letter of introduction could not undersatnd what I said. At last a pleasant-looking young nurse appeared, dressed much as English nurses are in a blue merino dress and white cap and apron, linen collar and cuffs. She invited me to follow her, and led the way upstairs.

The staircase is wide, and I noticed that the steps, which are low and broad, were covered with zinc, which has the advantage of being clean, noiseless, and durable, though I should fancy it would he rather slippery when wet. The floors both of the passages and of the wards, are parquetted in wood of two colours. On one side of the long passage are windows corresponding to those outside, so that anyone passing along the passage, can see into the ward. There are a great many of these windows, and they do not appear to have any curtains or blinds. This arrangement may be clean and light, but it destroys any appearance of privacy.

The nurse took me through the men's medical and women's surgical wards. All are high, light, and airy, but there are no blinds to the large windows. They are shielded only by thin washing curtains.

We went next to the theatre. My guide apologised for it not being very tidy, as there had been an operation that morning, and the nurses had not quite finished putting it straight, but it looked beautifully clean. The theatre is very nice and complete, though I thought the operating tables, of which there are two, not very up to date.

There were three or four young nurses cleaning up in a sort of ante-room off the theatre. They wore large white linen overalls something like those affected by artists, made high up to the neck, with large sleeves gathered into a band at the wrists, and fastened round the waist by a belt of the same material. It is not a becoming costume, but very sensible and serviceable, and worth imitating for proba- tioners when they are doing rough work. All the older nurses wore the same dress as my guide.

While I was looking round the room I saw the surgeons' white linen aprons hanging on hooks, and made the nurse laugh by telling her that in England the nurses call these "butchers' aprons"—a joke which she translated for the benefit of the probationers, and it seemed to amuse them very much.

My guide's English was not very fluent, so I could not ask the many questions which I should have been glad to put to her. She told me that she was nursing an English lady in one of the paying wards, and that she had just completed her three years' training. As she was in a hurry to get back to her patient I did not like to detain her longer, and took my leave, having spent a very interesting time and obtained a good general idea of a Dutch hospital containing over 300 beds.

### Central Midwives Board.

A meeting of the Central Midwives Board was held at Caxton House on Thursday, July 26. Dr. Champneys was in the chair, and there were also present Miss Paget, Mrs. Latter, Dr. Dakin, and Mr. Parker Young.

### The New Rules.

Some surprise was occasioned by the Chairman's announcement that the new rules which are being framed will not come into force at present. It has been decided that the operation of the existing rules shall be extended for another six months after the date of expiry, which will be August 12. It has also been decided to remove the training in Poor-law institutions from the jurisdiction of the Central Midwives Board, and they will in future be dealt with solely by the Local Government Board. One or two slight amendments were proposed, the most important being that it should be the duty of midwives to notify every birth in their practice, with the names of both parents of every child thus notified. Mr. Parker Young entered a strong protest against the extension of the present rules and the refusal of the Board to approve any Poor-law institutions as training schools.

### The Fees of Medical Men.

A letter was then read from Miss Hart, a certified midwife, respecting the payment of the fees of medical men called in by midwives. She stated that in the majority of cases in her own practice she had either had to pay the doctor herself or he had gone without his fee. She asked whether it could not be arranged that the parish doctor should be called in and his fees paid afterwards by the Guardians. The Board agreed that this could be done, and the Guardians would have power to pay the fees if their own medical officer were called to the assistance of a midwife.

### Resignation of a Lady Inspector.

A letter from two certified midwives approved by the Board for the purpose of signing Forms III. and IV., suggesting the appointment of another teacher in their neighbourhood was, after some discussion, referred to the Standing Committee.

The Secretary informed the Board of the resignation of one of the Lady Inspectors, who stated in her letter that it took nine hours to cover twenty-six inspections and another hour to fill up her report.

Keeping the Register.

The report of the Standing Committee dealt chiefly with correspondence on minor matters, and the report of Dr. Clapham, on the inspection of the register of cases kept by a midwife approved by the Board for the purpose of signing the certificates of attendance on cases, and attendance during the lying-in period. The register appears to have been imperfectly kept, and fails to show the name of the pupil to whom the case was entrusted. The Board agreed to the recommendation of the Committee that midwives approved by the Board for the purpose of training pupils shall insert in their registers under the heading "Remarks" the name of the pupil attending each case, and whether she delivered or nursed, each entry to be signed by the midwife, and the register to be open at all times for inspection by an officer of the Board.

The Certificates of Attendance.

On the motion of Miss Paget it was agreed that the Secretary be requested to report to each Board the number of suspensions that have been notified since the last meeting, together with the places notifying and the reasons given for suspension. Also that the number of suspensions for each year since the passing of the Act be presented to the Board. The Chairman moved that the Examination Schedule be amended by a declaration by the candidate of the truth of the four certificates contained therein; and that it be a condition of approval for the purpose of signing the certificates of attendance upon cases and during the lying-in period that the midwife keep a register of cases showing the name of the pupil attending each case, and whether delivering or nursing, and that the register be open to inspection at all times by an officer of the Board.

Both these motions were carried, and the proceedings closed with the fixture of the date for the next meeting for October 4.

Rule D. 1.

The following is a copy of the proposed Rule D. 1, with the additions suggested by the Privy Council in italics :—

"I. No person shall be admitted to an examination unless she produces certificates that she has undergone the following course of training, namely : (1) She must, under supervision satisfactory to the Central Midwives Board, *or, where the course of training has been undergone in a Poor-law institution, to the Local Government Board,* have attended and watched the progress of not fewer than twenty labours, making abdominal and vaginal examinations during the course of labour and personally delivering the patient (Schedule, Form III.). (2) She must, under supervision satisfactory to the Central Midwives Board, *or, where the course of training has been undergone in a Poor-law institution, to the Local Government Board,* have nursed twenty lying-in women during the ten days following labour, except in special cases in which a shorter period has been sanctioned by the *Central Midwives Board* (Schedule, Form IV.). The certificates as to (1) and (2) must be in the form prescribed by the Central Midwives Board, and must be signed by one or more of the following : (*a*) A registered medical practitioner; (*b*) the matron, being a certified midwife, or the chief midwife, being a certified midwife, of an institution recognised by the Board as a training school under the conditions provided in the Act; (*c*) a certified midwife approved by the Board for the purpose; (*d*) *where the course of training has been undergone in a Poor-law institution, a certified midwife attached to such institution and holding a qualification in midwifery satisfactory to the Local Government Board.* (3) She must have attended a course of instruction in the subjects enumerated in Rule D. 4, extending over a period of not less than three months, and consisting of not fewer than fifteen lectures. The certificate as to (3) must be in the form prescribed by the Central Midwives Board, and must be signed by one or more registered medical practitioners recognised by the Board as teachers, *or, where the course of instruction has been undergone in a Poor-law institution, by the duly appointed chief medical officer of the institution, or other medical practitioner authorised by the Guardians with the approval of the Local Government Board to give such instruction* (Schedule, Form V.)."

### Queen Victoria's Jubilee Institute for Nurses.

Her Majesty Queen Alexandra has been graciously pleased to approve the appointment of the following to be "Queen's Nurses," to date July 1st, 1906 :—

England and Wales.—Annie Elizabeth Evans and Annie Pierce-Jones, district training at Bermondsey; Cathlin Cecily du Sautoy, Margarete Egestorff, and Ada Morgan, Bloomsbury; Kate Mary Butler, Rose Emily Merrett, and Lucy Sophia Price, Brighton; Janet Scott, Burnley; Jennie Hughes, Camberwell; Mildred Dunn, Elizabeth Helen Gore-Hickman, Sarah Elizabeth Griffith, and Dorothy Jones, Cardiff; Janet Gibb, Gateshead; Ethel Lovelace, Homer-Mole, Gloucester; Matilda Stone; Jane Marion Holbrow, Huddersfield; Agnes Caine, Liverpool (Overton Street); Clara Ockleston, Liverpool (Shaw Street); Christina Fulton, Manchester (Ardwick Green); Charlotte Duncan Campbell, Sarah Mary Lizzie Hoad, Margaret Howells, and Kate Florence Young, Portsmouth; Grace Hunt and Mary Stevenson, Salford; Katherine Hall and Eva Winifred Bessie Lea, Shoreditch; Bertha Eunice Bennett, Warrington; Mary Anne Griffiths, Westminster.

Ireland.—Florence Susannah Moore and Mary Teresa Moylan, district training at St. Lawrence's Home, Dublin; Elizabeth Graham Campbell, Madeline Naylor, and Frances Alice Dania Thomas, St. Patrick's Home, Dublin.

Scotland.—Margaret Ann Green, district training at Dundee; Molly Elizabeth Black, Elizabeth Agnes Henny, Agnes Allan Kelly, Flora Macdonald, Jessie Stewart Mackenzie, Mary Ann Mitchell, Margaret Morrison, Annie Murray, and Mary Helen Watt, Edinburgh; Janet Barclay Calder and Jeanie Gordon, Glasgow.

### State Registration.

### MEETING AT EDINBURGH.

A well-attended and representative informal gathering of matrons and nurses from Edinburgh and the neighbourhood was held in Edinburgh on Monday last. Amongst those present were Miss Guthrie Wright, Miss Wade, Miss Sandford, Miss Milligan, Miss Lamont, Miss Cowper, Miss Wise, Miss Edwards, and Miss Shannon. Miss E. S. Haldane took the chair, and conveyed the regrets of Miss Louisa Stevenson and Miss Burleigh, who were unavoidably absent. Miss Haldane also referred to the encouraging resolution passed in London, July 27, by the annual representative meeting of the British Medical Association, agreeing with the Select Committee on the need of State registration for nurses, and that the medical and nursing professions should be adequately and directly represented on a general council. Miss Amy Hughes gave a short account of the movement, explaining the importance of the principle embodied in both Bills. Unless they made their views known they might find the question settled for them and not on the lines they expected. A discussion followed, opened by Miss Guthrie Wright, and an interesting meeting terminated by a hearty vote of thanks to Miss Spencer for her kindness in allowing the meeting to be held in the Royal Infirmary.

### Appointments.

[No charge is made for announcements under this head, and we are always glad to receive and publish appointments. The information, to insure accuracy, should be sent from the nurses themselves, and we cannot undertake to correct official announcements which may happen to be inaccurate. It is essential that in all cases the school of training should be given.]

Borough Isolation Hospital, Devonport.—Miss Jessie Baker has been appointed matron. She was trained at Stanley Hospital, Liverpool, where she has since been sister of the out-patient department and night superintendent. She has also been charge nurse at Mill Lane Hospital, Liverpool; charge nurse at St. John's Hospital, London; and superintendent of nurses at the Borough Sanatorium, Brighton.

Brixton District Nursing Association (Affiliated to Queen Victoria's Jubilee Institute).—Miss Florence W. Pritchard has been appointed superintendent. She was appointed Queen's nurse July 1st, 1897, and has since been Queen's nurse at Rushten, Chatham, Kensington, and Battersea. She has lately held the post of Inspector for Wales under the Institute.

Bury District Nursing Association (Affiliated to Queen Victoria's Jubilee Institute).—Miss Alice Jackson has been appointed superintendent nurse. She was appointed Queen's nurse July 1st, 1894, and has worked since that date under the Leeds District Nursing Association.

Kingston Victoria Hospital, Kingston-on-Thames.—Miss D. Dunn has been appointed matron. She was trained at Warneford Hospital, Leamington Spa, and has since been matron of the Cottage Hospital, Pershore. She has also done Army nursing.

St. Clement's Midwifery Training School, Fulham, S.W.—Miss Marian G. Bond has been appointed Sister. She was trained at the Chorlton Union Hospital, Manchester, and has since been district nurse at Ardwick Green, Manchester; charge nurse at the Clayton Vale Hospital, Manchester, E.; and district midwife in Wolverhampton.

St. Ives (Hunts) Union Infirmary.—Miss A. E. Longworth has been appointed head nurse. She was trained at Brownlow Hill Infirmary, Liverpool, and has since been temporary sister at Birkenhead Union Infirmary, and night sister at Chesterfield Union Infirmary. She has also done private nursing on her own account, and she holds the certificate of the Central Midwives Board.

South-Eastern Hospital, New Cross, S.E.—Miss Lillian M. Simpson, Miss Eliza Hothersall, Miss Catherine S. J Pugh, Miss Beatrice Wright, Miss Beatrice Wilsam, Miss Annie Latchford, Miss Bertha Garman, Miss Ethel M. Joyce, Miss Constance B. Mynill, Miss Lilian Raper, Miss Emily E. Rustin, Miss Emily Moore, Miss Laura Strickland, Miss Ethel Breakenridge, Miss Harriet Gimson, Miss Edith Bernard, Miss Edith Mustin, Miss Catherine S. Thomas, and Miss Rose D. Pratt have been appointed charge nurses. Miss Simpson was trained at the General Hospital, Birmingham, and has since been assistant matron at the Children's Hospital, Athens. Miss Hothersall and Miss Pugh were trained at the Royal Victoria Hospital, Belfast. Miss Wright and Miss Wilsam were trained at the Wandsworth Infirmary, London; Miss Wright has since been at the Victoria Hospital, Winchester. Miss Breakenridge and Miss Rustin were trained at the Fir Vale Infirmary, Sheffield. Miss Breakenridge has since been sister at the Infirmary, Gravelly Hill, and at the Union Hospital, Newcastle-upon-Tyne. She has done private nursing and holds the certificate of the Central Midwives Board. Miss Rustin has since been sister at the Fir Vale Infirmary, Sheffield. Miss Latchford was trained at the Infirmary, Nottingham, and has since been sister at the Isolation Hospital, Menston, in Wharfedale. Miss Garman and Miss Mustin were trained at the Lewisham Infirmary, London; Miss Mustin has since been sister at the Croydon Infirmary. Miss Joyce was trained at Guy's Hospital, London. Miss Mynill was trained at the Central London Sick Asylum, and has since been at the Nightingale Nursing Home, Southsea. Miss Raper was trained at Kensington Infirmary, and has since been at the Fever Hospital, East Ham. Miss Moore was trained at the Infirmary, West Ham, and has since been at the City Hospital, Birmingham. Miss Strickland was trained at the East Dulwich Infirmary, and has since been at the Somerset Hospital, Cape Town. Miss Gimson was trained at the Crumpsall Infirmary, Manchester, and has since been sister at the New Infirmary, North Evington, Leicester. Miss Bernard was trained at the Poplar and Stepney Sick Asylum, and has since been charge nurse at the Parochial Hospital, Dumbarton. Miss Thomas was trained at the Royal Free Hospital, London. Miss Pratt was trained at the Middlesex Hospital, London, and has since done private nursing.

Warrington District Nursing Association (Affiliated to Queen Victoria's Jubilee Institute).—Miss Martha Reeve has been appointed superintendent. She was appointed Queen's nurse July 1st, 1904, and has since worked as district nurse at Matlock, and as superintendent nurse of the Brixton District Nursing Association.

### Everybody's Opinion.

[Correspondence on all subjects is invited, but we cannot in any way be responsible for the opinions expressed by our correspondents. No communication can be entertained if the name and address of the correspondent are not given as a guarantee of good faith, but not necessarily for publication. All correspondents should write on one side of the paper only.]

### THE PENSION FUND AND SICK PAY.

"Belfast" writes: I am writing to express my appreciation of the Nurses' Pension and Sick Pay Funds, and to tell you of the great benefit received from the latter during an illness from which I have just recovered. I only joined the Royal National Pension Fund last February, and was taken ill exactly a month later. I have received sick pay during the entire time of both illness and convalescence (fourteen weeks in all), and am now well enough to begin work again. I write this as I think many nurses have the idea that they cannot receive sick pay until they have been policy-holders for several months, but this letter will prove the contrary.

### MENTAL CASE OR CHRONIC INVALID.

"Private Nurse" writes: Some time ago I went to Brighton to nurse a lady pregnant three months. One day she had pain and sent for the doctor. He assured her that it was nothing more serious than flatulence, but she made up her mind that she was going to have a miscarriage, and insisted on staying in bed. I was with her for a month, and she would not get up. During the month they had to change lodgings, and she had an ambulance from London to remove her a few yards. On leaving Brighton altogether another ambulance was ordered, and took her direct to her London home, and she was at once put under the care of her own medical man. After a lengthy examination he told her that everything was perfectly natural, and that if she would only lead a natural life she would probably have a nice healthy baby. From September to February she never went outside her door, and hardly got up from her bed. I used to go in and see her from time to time. On February 3 the child was born. The labour was quite natural and the child healthy. She was a very good patient, but still behaved herself as an invalid. At the end of three weeks the doctor told her that she could get up. She flatly refused to do so. The doctor then left her entirely in my care. I stayed on for six weeks, and then told her there was absolutely nothing I could do, so, amid tears and lamentations, I left, and I have not seen her since. I often wonder if she has even got up yet, and whether she has developed into a mental case or merely become a chronic invalid.

["Private Nurse" records a condition not uncommon during the puerperium, though by no means restricted to that state, or, indeed, to the female sex. Sir Wm. Gull was once consulted on just such another case. His diagnosis to the practitioner in attendance was, "The disease your patient is suffering from is not so much that she is ill but that she thinks she is ill, and we must not treat the disease but the '*thought*' of the patient. If that is done successfully all will soon be well."—Ed. The Hospital.]

### Presentations.

Kent Nursing Institution.—At the last committee meeting of Kent Nursing Institution, Tunbridge Wells, Miss Mottram, lady superintendent, was presented by the Dowager Countess of Aylesford with a silver-gilt medal and cheque in recognition of her eight years' devoted service in connection with the Institution.

### The Nurses' Bookshelf.

How to Become a Nurse: The Nursing Profession, How and Where to Train. Being a Guide to Training for the Profession of a Nurse, with Particulars of Nurse Training Schools in the United Kingdom and Abroad, and an Outline of the Principal Laws affecting Nurses, etc. Edited by Sir Henry Burdett, K.C.B. New and revised edition. (London: The Scientific Press, Limited, 28 & 29 Southampton Street, Strand, W.C. Price 2s. net; by post 2s. 4d.)

This useful little book has now reached its seventh edition, a fact which speaks eloquently for its usefulness. It is especially useful to the nursing aspirant, who would save herself and others much trouble if, instead of writing to the ever-busy matron of an institution, she got hold of this book, read the chapter telling of the common requirements of all nursing schools, and then, if she was prepared to comply with these, went on through the pages of the volume until she met with the institution which seemed best to suit her needs. And, on the other hand, matrons, who are pestered with endless communications from young women who may or may not prove suitable, would save themselves much trouble, and probably give a more satisfactory answer to the queries put to them, if they simply referred their correspondents to the pages of "How to Become a Nurse." For nurses who have completed their training the book is a convenient medium of information regarding the institutions where their acquirements are likely to obtain a position for them, the remuneration they may expect, and the conditions under which they will have to work. The list of foreign institutions is a great convenience for nurses who, for one reason or another, wish to go abroad, but are hampered by not knowing whether or not it is possible for them to earn their living in another part of the world. Of importance to all is the chapter dealing with the law as nurses are affected by it. On the one hand nurses are occasionally cheated out of their rightful earnings, and, on the other, they sometimes make claims which would never be allowed in law, and waste their money in useless litigation, when a little knowledge of their exact position would save trouble all round. As in the absence of a medical man, a nurse may occasionally be required to notify infectious diseases and see to the registration of births and deaths, it is important that she should know what is expected of her, and here she will find all the necessary information. In fact "How to Become a Nurse" is a *vade-mecum* which no nurse can afford to be without.

### TRAVEL NOTES AND QUERIES.

By our Travel Correspondent.

Belgium for a Fortnight (Queen's Nurse).—If you are only staying for two or three days in each place, pensions are of no use to you. So I give you the names of very moderate hotels. Ask always for rooms on the third floor. Take early coffee and rolls and late dinner in the hotel, and get mid-day lunch frugally in a confectioners. At Bruges, Hôtel Panier d'Or, in the Market Place; at Ghent, Hôtel Aux Armes de Zealande; or Hôtel d'Allemagne, both in the Marché Aux Grains. At Brussels, Hôtel de la Cathédrale, 17 Place Ste. Gudule, or Hôtel de Bavière, or Hôtel du Rhin, both in the Rue de Brabant. At Dinant, Hôtel des Familles. This is the cheapest; but, if full, go to Hôtel Tête d'Or.

Several Correspondents (E. Y. M.), (Jem), (Westeria), (A. L. W.).—Many thanks for addresses and recommendations. All will be filed for future use.

Autumn or Spring in the Black Forest (Inquirer).—The autumn is very beautiful there, but, of course, you have the disadvantage of shorter days and longer evenings. If your time is limited, I think you would do well to go to Freudenstadt *via* Heidelberg, and stay at the Schwarzwald Hotel. Money is reckoned in marks which corresponds with our shilling. Terms about six marks in August; less later on. There is a cheaper place if your stay there is longer than a week. Address Pension Villa, Margaretha. Terms five marks. Hornberg makes a good centre. Stay there at Hôtel Baer; five to six marks. At Venice, Hôtel Pension Kirsch on the Riva Degli Schiavoni, from seven lire; or Hôtel La Calcina, 782 Fondamentadella Zattere.

Pension at Havre (Cum Vino Spero).—I know nothing there that I can recommend. Living is dear, and not very pleasant. Dieppe is better, but still dear. Hôtel Soleil d'Or 4 Rue Gambetta, will charge you 8 frcs. per day. At Fécamp, Hôtel du Chariot d'Or, from 7 frcs., but it is not a nice place. Brittany is cheaper. Mrs. Dyott, Villa Olga, St. Malo, would take you on much lower terms if her house is not full; or Madame Pallot, Maison Matthias, St. Servan. You have left your inquiries far too late, I fear, for me to help you much.

A Holiday in Belgium (M. T.).—You must not expect an answer always in the next issue. Why did you not write to me earlier? You give me no idea of what you really want. Had you told me how long your holiday was to last, and what you could afford to pay, I could have been of real help. As it is I am quite in the dark. If your stay in each place is short, pensions are of no use to you; visitors are not taken for only two or three days. At Brussels go to Hôtel de la Cathédrale, close to Ste. Gudule, Hôtel de Bavière, or Hôtel du Rhin; these last two are in the Rue de Brabant. At Bruges, Hôtel Panier d'Or in the Market Place is very pleasant. At Ghent, Hôtel d'Allemagne, Marché aux Grains, or Hôtel aux Armes de Zeelande, same address. At Antwerp, Hôtel du Commerce, Rue de la Bourse, or Hôtel Grand Mirvir, 56 Vieux Marché. All these are reliable, but, being very moderate in price, you must not expect much luxury.

Rules in Regard to Correspondence for this Section.—All questioners must use a pseudonym for publication, but the communication must also bear the writer's own name and address as well, which will be regarded as confidential. All such communications to be addressed "Travel Correspondent, 28 Southampton Street, Strand." No charge will be made for inserting and answering questions in the inquiry column, and all will be answered in rotation as space permits. If an answer by letter is required, a stamped and addressed envelope must be enclosed, together with 2s. 6d., which fee will be devoted to the objects of "The Hospital" Convalescent Fund. Ten days must be allowed before an answer can be published.

### A Book and its Story.

### AN UNSETTLED POSSESSION.*

Mrs. Campbell Dauncey, in her brightly written letters, gives an interesting description of the Philippines and the Filipinos under American rule. Nine months in that "uneasy land" sufficed to make her acquainted with many of the questions arising out of the political situation. During her sojourn she wrote frequently to friends at home giving her first impressions of scenes and people while the impressions were still vivid. The political situation looms large in conversations, in the daily life, and in descriptions of that life. In proof of this Mrs. Dauncey prefaces her book with an explanation of the impossibility of leaving it out in any book on the Philippines. She writes with an open mind, holding no brief for the Americans or the Filipinos, her object being to convey a true and unbiassed impression of what she saw of the Philippines as they are.

Life moves on primitive lines in these islands, as the following account of a house-hunt shows: "We are busy house-hunting, which is a tedious and toilsome business, as there is no such institution as a house agency. You allow a rumour to get about that you want a house, and then people tell other people to let you know where an empty house is to be found. Then you go off and find the house—a matter usually of infinite difficulties, and sometimes quite impossible, as the Filipino cabdrivers do not know the names of the streets, or the numbers, or the names of the people. Having found a house you set to work to find the owner of it, who is probably playing cards; and when he or she appears, you ask—and this is quite necessary—if the house is to let, for the board does not signify much. . . . When the house is really to let, you ask the rent, and whatever the answer is you throw up your hands in horror and declare it is very dear, and that you will give half—calling assorted saints to testify all the drawbacks which make the house unfit for human habitation at any price." Although trade is very bad, and many houses are standing empty, the rates and taxes are appallingly high, and the rents, too, in consequence, and out of all proportion to the accommodation offered. But the curious half-bred Filipino, lazy and supercilious, prefers to keep a house empty, and say it cannot be let under 100 dollars a month, than accept 50 for it and live in comfort. The houses are all two-storied—the upper part of wood and the lower of stone or concrete. The floors are of the hard, dark native wood, kept bright by the native servants, who polish them with petroleum pads on their feet. The walls are of wood, in partitions painted white or green, and in the corners of the room appear the big tree trunks to which the house is locked—sometimes encased in a wooden cover. The houses are tied together with a strong fibrous vine, so as to allow sufficient play for earthquakes, which are so frequent in the Philippines as to hardly call for comment. The windows are merely sliding shutters formed to close against rain and sun. For the rainy season, others, composed of small square panes filled with a very fine white pearl oyster shell, are used. No chimneys or fireplaces can be used on account of the monsoons, during which the houses could not be kept dry. From the general description given the Philippine houses must be quite pretty and lend themselves to decoration. Tall trees in Chinese stands of blue and green earthenware are a favourite combination, and when orchids of rare and delicate hue, which grow in profusion, are added, a charming contrast to the white paint and brown walls is supplied. The climate of the Philippines is summed up as follows: "Four months dry and cool; four months dry and hot; four months wet and hot. That is the climate over most of the Philippine Islands; but it varies in sequence in different places—areas is a better word—and on the Pacific seaboard the seasons are quite reversed, so that it is rainy there when it is dry here. By rain and dry, however, I gather that a great deal of drought, or a long steady rain is not meant, for all during the dry season there are heavy showers, and everything remains green; while in the wet season there are spells of fine weather." The evenings are described as charming, and the early mornings delicious. Dawn begins at half-past five, and from then until half-past eight the air is delightful; after that shade has to be sought and fans requisitioned to make it bearable. The winter goes on till March, and there is a steady increase of temperature till June, when the monsoon drifts to the south-west and the rainy season begins. The average temperature in the winter is 83 Fahrenheit. But the arrangements for comfort and coolness are those of a hot climate, and not such as we have at home, which makes a considerable difference. Among the great charms of the Philippines must be mentioned its sunsets, which do not vary very much, except in intensity of colouring. The author speaks of them as "eternally beautiful sunsets." She describes one seen from the balcony of the house. "The sky behind the palms in the distance was deep orange, fading into rose, and overhead into apple-green blue. We went through the house and out on to the azotea, and all the sky on that side was like a radiant pale amethyst, with a big bright moon rising—a great silver shield—through the lilac and rosy mist; the waters a deep sapphire blue; and Guimaras (an island) a brilliant green outline dividing the sea and sky. The tide came up to the wall at the end of the garden. . . Words fail me to describe the scenery! The blue and green of the sea, the mauve and rose lights, reflected on the Guimaras from the brilliant sunset behind us over the Danay Mountains, were like some wonderful picture wrought in amethysts and sapphires and exquisite enamels, while all along the shore-line the groves of palm trees glowed in a strong light like a border of emeralds set in golden sand." The Filipinos are a very mixed race. "Spanish, Chinese, European, every nation under the sun" is mixed with the native race. In appearance they are "a funny little people, the women averaging well under five feet, with pretty slender figures and small hands and feet. The original race was a little fuzzy-headed, black people, remnants of which are still to be found in the mountains and smaller islands. . . . The average Filipino is the same everywhere. . . . The Filipinos are very fond of music and dancing. They are a singularly happy people when unspoilt by the advantages of civilisation. . . . One never hears them quarrelling; they are kind to their children. . . . To watch a group of poor people is always a pleasure. Indolence and procrastination, as with other tropical races, seems to be their bane. An American hospital was being erected during the author's stay." The funds for its erection had been collected; "and we have all contributed by request. . . . This remarkable building is slowly rising from a pile of beams and planks to certain lengths. I say it is remarkable because the hospital has evidently been designed in America by someone who has never heard of the Philippines, for the main supports are being made of . . . quite slender posts of Oregon pine . . . which is about as much good as cardboard against insects, typhoons, earthquakes, and so forth." But if the superstructure was slight for the purpose, the wards were even more so, and were formed of screens of nipa—a befuco matting. In consequence: "the hospital was the laughing stock of the town and the subject of many rather acid jests on the part of those who had contributed to such a monument of folly . . . all so hasty, so shoddy." The author's description of this far-away and little-known part of the world is a notable contribution to the literary handbooks that now are so general. Illustrations are not wanting to add to the attractions of this interesting book.

* "An Englishwoman in the Philippines." By Mrs. Campbell Dauncey. (John Murray. Illustrated. 12s. net.)

### Notes and Queries.

**REGULATIONS.**

**The Editor is always willing to answer in this column, without any fee, all reasonable questions, as soon as possible.**

**But the following rules must be carefully observed.**

1. **Every communication must be accompanied by the name and address of the writer.**
2. **The question must always bear upon nursing, directly or indirectly.**

**If an answer is required by letter a fee of half-a-crown must be enclosed with the note containing the inquiry.**

*India.*

(211) Where can I obtain further information respecting the organisation of a staff of English nurses for Indian people?—*Claremont.*

The scheme is still in its early stages, but a letter addressed to Lady Minto, Viceregal Lodge, Simla, Calcutta, is sure to receive attention. See our issue of July 21 for details.

*Holiday Home.*

(212) Can you tell me of a Holiday Home where I can go for complete rest and change for a few months, paying a small sum?—*Leytonstone.*

There is a Home of Rest at Lansdowne, Cavendish Road, Felixstowe, Suffolk, 5s. to 15s. weekly. Address Miss Andrews. Also write to Miss Robarts, St. Mary Convalescent Home, Beach Avenue, Birchington-on-Sea. Moderate fees. Charges at Crescent House, Marine Parade, Brighton, 8s. to 12s. a week. Write to Mrs. Donkin.

*Tuberculosis.*

(213) Can you tell me of a sanatorium where an orphan girl in the early stages of consumption can be received free?—*R. M.*

As you are in London, write first to the Secretary of the Mount Vernon Hospital, Hampstead. If unsuccessful, try the Royal West of England Sanatorium, Weston-super-Mare. Charge 10s. 6d. a week. Nearly every sanatorium has a slight charge. Kelling Sanatorium, Holt, Norfolk, has some free beds.

*No Premium.*

(214) Can you tell me of a hospital in Yorkshire where I could train without premium?—*Scarborough.*

The Halifax Infirmary pays £6 the first year to probationers and the Hull Royal Infirmary pays £8 the first year.

*Philadelphia.*

(215) Can you tell me about the Philadelphia Hospitals? I expect to be in America next spring, and wish to become a nurse there.—*St. Andrews.*

There is an excellent training school for nurses at the Philadelphia Hospital (1,500 beds), and there is also the Philadelphia Women's Hospital and a hospital at Penna.

*Salisbury Treatment.*

(216) Can you give me the name of a book describing the Salisbury Treatment?—*Fermain.*

"What Must I do to get Well?" 6s. 4d. post free from The Scientific Press, 28 Southampton Street, Strand.

*Quarantine.*

(217) Is it a matron's duty to see that a nurse has reasonable off-duty time to disinfect in a fever hospital, and to take outdoor exercise without absolutely leaving the hospital?—*Nurse A. M.*

It is a matron's duty to see to the physical well-being of her nurses, and that all rules of an institution are carried out.

*How to Become a Nurse.*

(218) I want to be a nurse in a fever hospital. How should I go about it?—*Reader.*

Get "How to Become a Nurse." The Scientific Press, 28 Southampton Street, Strand, 2s. 4d., post free.

*Stammering.*

(219) Can you tell me how to be cured of a slight impediment in my speech? I wish to be a nurse.—*Loughborough.*

This is a medical question which you had better refer to your doctor.

**Handbooks for Nurses.**

| | Post Free. |
|---|---|
| **"How to Become a Nurse: How and Where to Train."** | **2s. 4d.** |
| **"Nursing: its Theory and Practice." (Lewis.)** ... | **3s. 6d.** |
| **"Nurses' Pronouncing Dictionary of Medical Terms."** | **2s. 6d.** |
| **"Complete Handbook of Midwifery." (Watson.)** ... | **6s. 4d.** |
| **"Preparation for Operation in Private Houses."** ... | **0s. 6d.** |

**Of all booksellers or of The Scientific Press, Limited, 28 & 29 Southampton Street, Strand, London, W.C.**

### For Reading to the Sick.

LOST AND FOUND.

Jesus my Shepherd is,  
'Twas He that loved my soul,  
'Twas He that washed me in His Blood,  
'Twas He that made me whole;  
'Twas He that sought the lost,  
That found the wandering sheep,  
'Twas He that brought me to the fold,  
'Tis He that still doth keep.

*Dr. Bonar.*

In each soul which is won to God, Jesus "sees of the travail of His soul, and is satisfied"; each lost sheep which is found Jesus lays on His shoulders rejoicing, and says to His friends, the blessed angels, "Rejoice with Me, for I have found My sheep which was lost." Such joy there is over every one won to the faith, over every one who, having forfeited his baptismal grace, is restored by true repentance and conversion, so that he who was dead, again lives; such joy over every true confession, in which the soul, whose sins were scarlet, is washed white as snow in the Blood of Jesus, and Jesus says, "Thy sins be forgiven thee."—*E. B. P.*

Whatsoever may befall us, let us say, It is the Voice of the Good Shepherd. It is His rod and staff which smite and comfort me. This will convert all things into revelations of His nearness and of His compassion. If it be disappointment, perhaps we were too bold and confident, and there were in our course pitfalls and death. If it be long anxieties, perhaps we were settling down in this life with too full a rest. If our long anxieties have shaped themselves at length into the realities of sorrow, it was that we needed this for our very life, that nothing else would work in us His will and our salvation. Let us thus learn to taste and to see that He is with us. All things are His doing, and that is enough.—*H. M.*

So Thou also, Merciful Father, dost more rejoice over one penitent, and with much joyfulness do we hear with what joy the sheep which had strayed is brought back to the Shepherd's shoulder; and joy forceth to tears, when in Thy house it is read of Thy younger son, that he was dead, and lived again: had been lost, and is found. For Thou rejoicest in us and in Thy holy angels, holy through holy charity. For Thou art ever the same. The conquering commander triumpheth, yet had he not conquered unless he had fought; and the more peril there was in the battle so much the more joy there is in the triumph. The storm tosses the sailors, threatens shipwreck; sky and sea are calmed, and they are exceeding joyed, as having been exceeding afraid. Everywhere the greater joy is ushered in by the greater pain.—*S. Augustine.*

Recollect, O Love Divine,  
'Twas for this lost sheep of Thine,  
Thou Thy glory didst resign.

Judge of Justice, hear my prayer!  
Spare, O Lord, in mercy spare!  
E'er the Reckoning Day appear.

## Figures and Tables

**Figure f1:**
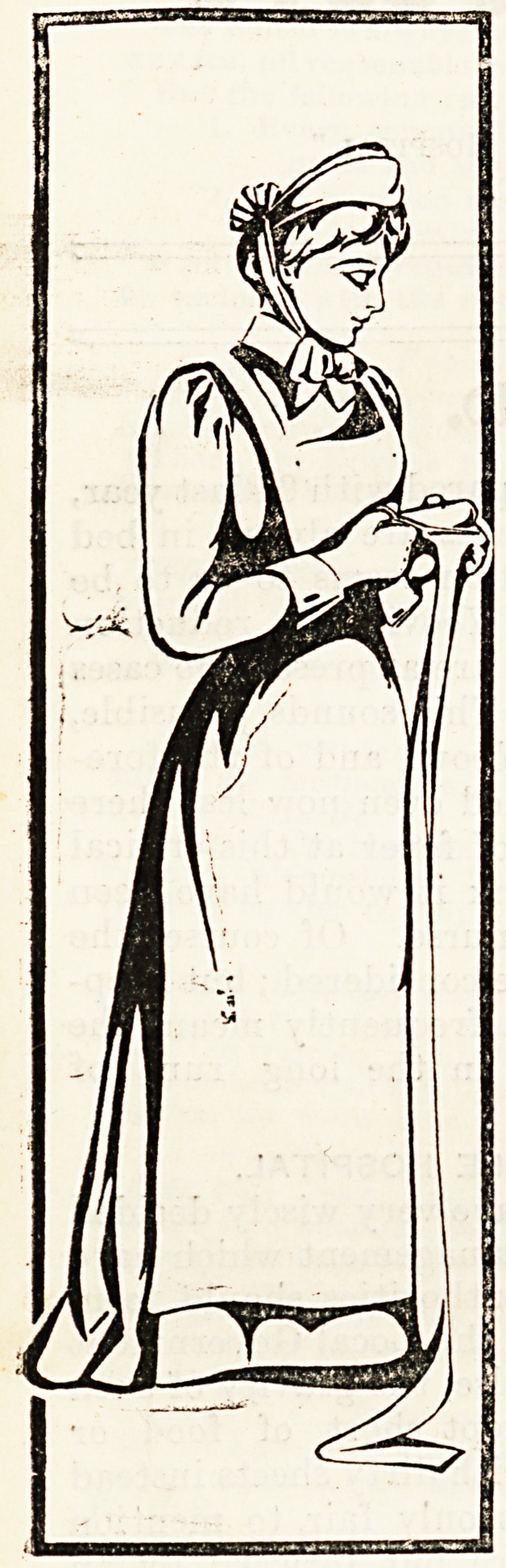


**Fig. 17. f2:**